# ‘For me it’s just the conversation:’ responsive feeding influences among early childhood educators

**DOI:** 10.1017/S1368980024001885

**Published:** 2024-10-04

**Authors:** Olga Levin, Jessie-Lee D McIsaac, Julie Campbell, Elizabeth Dickson, Melissa D Rossiter

**Affiliations:** 1 Early Childhood Collaborative Research Centre, Mount Saint Vincent University, Halifax, NS B3M 2J6, Canada; 2 Faculty of Education, Mount Saint Vincent University, Halifax, Canada; 3 Department of Child and Youth Study, Mount Saint Vincent University, Halifax, Canada; 4 School of Health and Human Performance, Dalhousie University, Halifax, Canada; 5 Department of Applied Human Sciences, University of Prince Edward Island, Charlottetown, Canada

**Keywords:** Behaviour change, Nutrition, Responsive feeding, Early childhood, Early learning and child care

## Abstract

**Objective:**

Early learning and childcare (ELCC) programmes play an important role in shaping children’s eating behaviours and long-term health by establishing a responsive feeding environment that encompasses not only mealtime behaviours but also extends to play activities and language used throughout the day. Despite their potential benefits, many ELCC centres do not consistently implement responsive feeding behaviours, facing challenges with organisational and behavioural changes within these environments. This study aims to identify influences on responsive feeding behaviours among early childhood educators prior to an intervention.

**Design:**

A qualitative study guided by the Behaviour Change Wheel framework and Capability Opportunity Motivation – Behaviour (COM-B) model. Semi-structured interviews and focus groups were conducted, recorded and transcribed verbatim. Thematic analysis was employed to identify themes, categorising them within the corresponding COM-B domains.

**Setting:**

Canada.

**Participants:**

Forty-one ELCC staff in various roles across eight centres from two provinces in eastern Canada.

**Results:**

Fifteen influences, spanning across all six domains of the COM-B model, were identified, highlighting gaps in educators’ knowledge and skills, varied approaches to food and feeding, and the interactions with children, parents, and co-workers on mealtimes dynamics. Additionally, costs, centre location and other physical resources emerged as enabling opportunities for responsive feeding behaviours.

**Conclusions:**

These findings offer a comprehensive exploration of the diverse factors influencing responsive feeding behaviours among educators, each varying in its potential for future behaviour change intervention.

The early years of children’s lives are characterised by rapid and profound development and eating patterns established in childhood can lead to lifelong behaviours. A diet that is varied and rich in nutrients supports the child’s cognitive development, optimal growth and immune system, while also shaping future food choices^(1,2)^. However, focusing on diet alone does not consider the impact of the eating environment, which also plays a role in how children view and approach food and feeding^(3,4)^. Children are born with the innate ability to recognise their hunger and satiety cues and self-regulate their food intake^(3,4)^. Through responsive feeding, caregivers can maintain this natural ability by providing prompt, emotionally supportive and developmentally appropriate responses to children^(5)^.

Responsive feeding is built on a foundational focus from active feeding^(2,6)^ and responsive parenting^(5,7)^ and includes a division of responsibility where caregivers decide when and what food will be offered and the child decides whether, and how much, they will eat from what is available^(8)^. When utilised, responsive feeding enables children to communicate their food-related needs, increases mindfulness to support self-regulation and promotes recognition of internal hunger and satiation cues^(5,9,10)^, leading to short and long-term health outcomes^(5)^. Conversely, controlling and pressuring feeding behaviours that override children’s hunger and satiety cues are associated with more food aversions and emotional eating, less consumption of fruits and vegetables and suboptimal weight trajectories^(1,11–13)^. Forms of pressure can include rewards (food and non-food) for eating, excess attention on trying food and labelling foods as healthy or unhealthy. These practices can hinder children’s ability to recognise their hunger and satiety cues and may lead to feelings of guilt or shame if they eat or do not eat certain foods.

Approximately half (56 %) of Canadian children under the age of 6 years are enrolled in licensed and unlicensed early learning and childcare (ELCC) programmes across the country^(14)^. Mealtimes constitute a substantial part of ELCC routines and as a result, early childhood educators (ECEs) have a profound influence on young children’s feeding environments^(3,15)^. ECEs facilitate responsive feeding by role modelling acceptance and consumption of new foods in a supportive environment that does not place pressure or coerce children to eat^(16)^. They do this by prompting children to recognise their hunger and satiety cues^(9,17)^ and using neutral language when discussing food^(18)^. A meal service that allows children to self-serve and select their portions from shared dishes placed on the table (often referred to as family-style) also enhances children self-regulation and serves as an opportunity for children and ECEs to eat together in a pleasant and relaxed atmosphere^(15)^. Furthermore, ECEs can extend their impact beyond mealtime by providing opportunities for children to explore, play with and learn about food without the pressure to eat^(19,20)^.

However, there is a gap between what is known about responsive feeding and implementation in ELCC programmes. Studies report perceived barriers to responsive feeding, such as believing that children serving themselves is impractical, messy, and leads to waste^(21)^. Additionally, there is a lack of training for educators on how to support children’s varied eating behaviours^(22,23)^. In one previous study, nearly half of the educators observed responded to refusal to eat with controlling behaviours, such as insisting and pressuring the child eat certain foods^(24)^. While educators generally have positive intentions during mealtimes^(9,25,26)^, they may resort to these controlling practices due to lack of trust in children’s ability to recognise their fullness cues^(24)^, the perceived effectiveness of pressure and coercive practices and the desire to please parents^(25)^. Other reasons include not wanting children to be hungry and reducing food waste^(26)^. Constraints on financial resources and time can also limit opportunities to create a responsive feeding environment^(27)^. There are few studies that take a comprehensive approach to understand the full spectrum of behavioural influences on responsive feeding, particularly to inform intervention^(28)^.

One prominent framework that provides a lens to consider responsive feeding is the Behaviour Change Wheel (BCW), which consists of three interacting layers (Fig. 1). The two outer layers are policy categories and intervention functions, while at the centre of the BCW are sources of behaviour, also known as the Capability Opportunity Motivation – Behaviour (COM-B) model^(29)^. For a behaviour to occur, one must have not only motivation but also capability and opportunity, both of which influence the motivation to engage in the behaviour. In the context of responsive feeding, capability refers to the caregiver’s psychological and physical ability to engage in responsive feeding behaviours, such as their knowledge and skills. Motivation is automatic (e.g. emotions and impulses) and reflective (e.g. alignment of their values), and opportunity includes the social and physical factors that prompt responsive feeding behaviours, such as peer support and physical resources. Altogether, a COM-B analysis of responsive feeding behaviour can highlight areas in need of change to later inform policy categories and intervention functions^(29)^. A recent systematic review using COM-B analysis identified responsive feeding enablers and barriers among parents, which mirror those experienced by ECEs, including specific feeding attitudes, beliefs, intentions and a lack of responsive feeding knowledge and skills^(30)^. Although recent studies have used the COM-B system to conceptualise nutrition behaviours in the community^(30–32)^, seldom has the COM-B framework used in ELCC programmes.

**Fig. 1:**
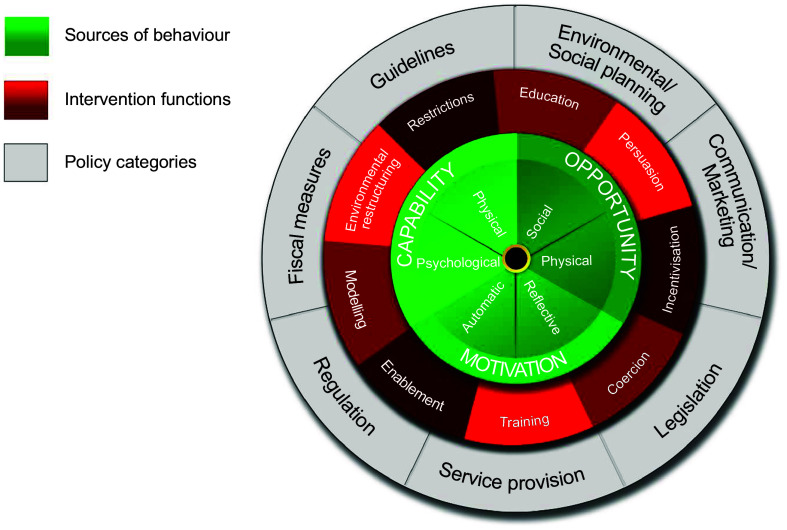
The Behaviour Change Wheel. Source: From Michie, S., Van Stralen, M.M. & West, R. 2011

CELEBRATE Feeding (*Coaching in Early Learning Environments to Build a Responsive Approach to Eating and Feeding*) was a feasibility study conducted in Nova Scotia and Prince Edward Island that worked directly with ELCC centres with the objective of enhancing responsive feeding in ELCC using the BCW as a guiding framework for intervention with ECEs. The purpose of this study is to apply the COM-B model to describe the influences on responsive feeding behaviours among ECEs in ELCC programmes prior to intervention.

## Methods

A Qualitative Description (QD) approach was used in this study to provide a rich description of the influences of responsive feeding from the perspective of ECEs^(33)^. QD allowed the researchers to remain close to the data throughout the analytical process^(33)^ to reflect components of the COM-B model. QD was applied within the lens of a social constructivist framework, which reflects the belief in the co-construction of ECEs’ knowledge of the influences on the feeding environment through their interactions with children, colleagues and families^(34)^. In the CELEBRATE Feeding intervention, the COM-B system provided a framework to consider these unique experiences to identify potential target behaviours to support implementation of responsive feeding. The COREQ checklist was reviewed and used to inform reporting of our qualitative methods^(35)^.

### Data collection

Centres in the Canadian provinces of Nova Scotia (*n* 5) and Prince Edward Island (*n* 3) were recruited through a public call for interest, sent via email, promoted on social media and detailed in a virtual information session. The participating ELCC centres were purposively selected based on their interest and capacity to participate in the intervention, while also ensuring a mixture of sizes and demographics. Forty-one ELCC staff from participating centres provided informed consent to take part in a semi-structured individual (*n* 25) and group interviews (*n* 4). Interview guides were developed by the research team to gather contextual information on the ELCC programme and staff experiences with food and feeding prior to the CELEBRATE Feeding intervention. Participants were asked about the team approach to food and feeding at their centres, their top priorities during mealtimes and the role of food outside of mealtimes. Most participants identified as women (87·8 %), were between the ages of 36 and 45 years (33·3 %) and held diverse roles at the centre, including classroom ECEs (*n* 32), directors (*n* 7), one inclusion coordinator and one cook (Table 1). The audio-recorded focus groups and interviews (∼20–40 min) were conducted in-person at the centres or virtually, between October and December 2022, by an Early Years Nutrition Coach (Registered Dietitian); participants received a $20 gift card for participation. The recordings were transcribed verbatim by a trained research assistant, then reviewed and de-identified by a second assistant. This project was approved by the University of Prince Edward Island and Mount Saint Vincent University’s Research Ethics Boards.

**Table 1:** Participant characteristics (*n* 41)

	*n*	%
Gender	*n* 33*	
Woman	29	87·8
Man	2	6·1
Prefer not to answer	2	6·1
Age	*n* 33*, *n*	%
< 25 years	6	18·2
26–35 years	5	15·2
36–45 years	11	33·3
> 46 years	9	27·3
Prefer not to answer	1	3·0
LGBTQ2+, yes, *n* (%)	1	3·0
Newcomer, yes, *n* (%)	5	15·2
Ethnicity	*n* 31*, *n*	%
European	12	38·7
Asian	6	19·3
Other	8	38·1
Prefer not to answer	3	9·6
Years ECE experience	*n* 33*, *n*	%
< 5 years	12	36·3
5–9 years	2	6·1
10–19 years	5	15·1
> 20 years	14	42·4
Highest completed education in early childhood	*n* 25*, *n*	%
None	3	12
Some coursework (i.e. level one certification)	2	8
ECE college certificate	4	16
ECE college diploma	14	56
Bachelor’s degree or beyond	3	12
Roles within the ELCC centre	*n* 41, *n*	%
Early childhood educator	32	78
Director	7	17
Other staff (e.g. inclusion coordinator, cook)	2	4·9
Main room(s) educators worked in	*n* 36†, *n*	%
Infant	4	12·1
Toddlers	10	21·2
Preschoolers	14	24·2
Mixed ages	6	15·1
School age	2	6

ECE, early childhood educator; ELCC, early learning and childcare.

* *n* differs as some participants did not complete the baseline questionnaire as part of the larger CELEBRATE Feeding project, as well as due to missing answers.

† Some educators covered multiple classroom, excluding directors.

### Data analysis

The transcribed interviews were analysed by the lead author (a master’s trained Registered Dietitian) in multiple stages with an emphasis on QD through a process of reflexive thematic analysis^(36)^. First, the researcher gained familiarity with the data by reading through the transcripts and identifying emergent codes through an inductive process. Concurrently, a deductive approach was employed to search for codes associated with the COM-B model. This dual strategy confirmed the depth and richness of the data for the subsequent COM-B analysis, while also remaining close to the social experiences of participants. Next, a more thorough line-by-line coding was conducted in MAXQDA 2022 (v.22..2..1) to enable a detailed analysis, exploring connections and relationships between codes. These codes were then grouped into themes and matched with COM-B domains. To ensure agreement on domain interpretation given varied definitions^(32)^, the COM-B domains and their definitions were reviewed through iterative discussions within a larger research team with expertise in dietetics and early childhood, including those that conducted and transcribed the interviews. The final themes were named and refined through ongoing discussions with the team.

## Results

The identified influences on participants’ responsive feeding practices are summarised within the six COM-B domains (Table 2).

### Capability

#### Psychological capability

##### Navigating feeding situations

Many participants reported a lack of confidence in applying knowledge and skills when feeding children who had difficulties with eating. These difficulties were described as an inability to sit at the table for mealtimes, extremely selective eaters and children refusing to eat or consuming large portions. One educator expressed their concerns about a specific child: *‘…so that’s a little guy that is probably the most difficult for me because there is so many aversions and I don’t know how to support him at school’*. In an attempt to manage these challenges, participants described non-responsive behaviours that may override children’s internal cues, such as pressuring children to try foods, or rewarding them with stickers or more desirable food (e.g. crackers). Several educators reported preventing certain children from eating or not providing subsequent servings if they believed the child ate too much. Conversely, some responsive behaviours were also noted in challenging feeding scenarios, such as ensuring familiar foods were served, employing role modelling techniques and not requiring children to remain at the table until they have finished eating all foods on their plates.

##### Balancing between pressure and encouragement

It was clear that many participants unknowingly applied pressure on children with the intention to encourage children to explore and eat a variety of food. One director explained their team approach to feeding: ‘*…we want to encourage them to try it, so everything will go on their plate, but we’re not forcing them to eat it’*. Educators also described subtle pressure, asking children to try just one bite, praising children in front of their peers for trying certain foods or commenting on the food’s health benefits. These behaviours constitute forms of pressure and interfere with the child’s autonomy to decide what and how much food to try. Some participants mentioned explicitly that they did not know how to encourage children to try their food without exerting too much pressure: *‘I don’t know when to push, to say “you should try it” or if I should just let themselves figure out what they want. I do struggle with that because I want to do it right’*.

##### Managing available food

Some participants expressed that they did not know how to let children eat what and how much they wanted while also ensuring there was enough food for all children. As one educator explained, *‘If they get to choose that they just want to eat strawberries the first round, I have a problem. Am I like “just leave some for your friends?”, I don’t know… that is a struggle to me’*. Further, participants identified the dynamic nature of mealtimes as a challenge, noting that children’s preferences and appetites change day to day and each educator may serve different portion sizes; thus, food quantities can be difficult to manage. In addition, it was found that there is limited to no support for nutrition and menu planning in ELCC centres, and the current provincial nutrition guidelines were described by participants as not comprehensive enough, outdated and lacked flexibility.

#### Physical capability

##### Communication competence

Participants’ ability to communicate with children during mealtimes and to understand their needs, including hunger and satiety cues influenced responsive feeding, although this theme was less commonly reported. Participants reported using sign language and images of the served foods as non-verbal ways to communicate with children who were younger or had language barriers. The ability to identify and correctly interpret body language was highlighted by participants as another way to understand and follow children’s hunger and satiety cues; one educator described: *‘…we go around the table… and we’ll be like “do you want another bite” and if they turn their head we don’t… we generally don’t encourage them to keep eating… when they’re done, they’re done*’.

##### Dietary restrictions and educator preferences

A few participants mentioned that their directors encouraged them to eat the food served alongside children, but their own dietary needs and food preferences were a barrier. Children with specific dietary needs also bring their own food, which was felt to change the meal dynamics. For example, one participant described a child bringing food that was perceived to be more desirable and the ECEs felt they needed to convince the rest of the children to eat from what is served.

### Motivation

#### Reflective Motivation

##### Healthy start/nourishment

Some participants expressed the belief that children should be exposed to diverse foods from a young age and rely on their internal cues for eating. As one director said: ‘*If we’re always telling children, “you’re done,” or “you need to eat more,” they’re not learning on their own what is enough, what isn’t enough, they’re not cueing into their being hungry or their being full’.* In other cases, participants tried to pressure children to eat in a certain order based on what they perceived was ‘healthier’ or ‘adequate’ for the child: *‘Well I’ll give you muffin after you eat your strawberries because you like them and I want you to have fruit too, not to just fill your belly with muffins’.*

##### Mealtime ‘isn’t just about food’

Participants also referred to mealtimes as a special and enjoyable time where children can laugh, bond with their peers and teachers, and grow their social-emotional connections. These participants mentioned that they focus on communication at the table and appreciate family-style meals. One of the directors shared their enthusiasm around mealtime: *‘I love to hear the educators and the children, they’re laughing and they’re having a great time, it never seems to be stressful…it’s a social time’.* This theme was found to be closely related to other responsive behaviours, such as avoiding pressure and modelling opportunities. Educators also indicated their own childhood mealtime experiences and upbringing (e.g. being forced to eat/clean the plate) as shaping their mealtime approach. Relatedly, some educators expressed disappointment that food is no longer a part of celebrations in ELCC settings like it was during their own childhood.

##### Ensuring intake

Conversely, for other participants, ensuring that children would not be hungry was their main priority during mealtimes. A few participants spoke about negative moods when children were perceived to be hungry, for example: *‘…if they [children] don’t eat lunch, they’ll be cranky in the afternoon. So, I think that might be the priority, get food into them’.* In some cases, this approach was associated with non-responsive feeding behaviours where participants put pressure on the children to eat or to try foods, as one participant said, *‘basically making sure that the meal is eaten’.* On the other hand, some participants with this approach also engaged in slightly more responsive behaviour, such as letting the child decide what they want to eat as long as they eat something.

#### Automatic motivation

##### Enjoyment & fun

Participants frequently emphasised the importance of creating a *‘fun’, ‘enjoyable’* and ‘*happy’* atmosphere for the children, which served as a motivating factor to make mealtimes more positive (e.g. creating a calm environment, dimming the lights and not pressuring children to eat when not interested or too tired). Participants also described children’s enjoyment of food-related activities outside of mealtimes, such as baking and pretend play in the mud kitchen, which seemed to motivate them to engage in these activities that supported food exploration.

##### Managing mealtimes and competing priorities

Some participants felt that mealtimes were just another routine task required; they expressed that they did not have a specific priority during mealtimes or adopted a more practical approach where they ensured adherence to hygiene and served the food. Others expressed competing priorities, including making sure that children were fed, tracking food consumption, preparing for the next scheduled routine, while also accommodating the breaks for ECEs. Managing priorities sometimes led to a rushed and hectic mealtime environment and made it difficult for ECEs to engage fully in responsive feeding behaviours. Allowing flexibility with the schedule, especially with snack time, and family-style meals were viewed as helpful by some participants to allow ECEs to engage in responsive feeding.

### Opportunity

#### Social opportunity

##### Intra- and interprofessional dynamics

Mealtime behaviours of ECEs were also influenced by each other and by other professionals. Similar feeding approaches, a shared mission, or educational backgrounds seemed to facilitate open communication and collaborative mealtime dynamics. Although many participants felt their team agreed on feeding behaviours, there were also challenges navigating differences in individual approaches. There was a strong sense of collegiality and respect among the participants, and ECEs noted discomfort in critiquing their colleagues if a behaviour did not align with their own or a perceived best practice. One participant pointed out: *‘There are other people in the centre… that do tend to do food rewards and it’s not something that I’ve opposed—like I’ve never been like “hey maybe we shouldn’t do that”’.* Another participant explained that they fear being disrespectful, especially if the reason for the behaviour is rooted in cultural beliefs. Additionally, relationships and collaboration across different roles within the centre may have also influenced responsive feeding. For instance, at some centres, the cook or director would engage with children and talk about food.

##### Child-educator dynamics

Certain child characteristics, such as age, were also found to influence the interactions and responsive behaviours. For instance, ECEs sometimes struggled to interpret infant hunger and satiety cues, which may have led to increased pressure or prematurely ending mealtime. Other educators felt it was easier to feed younger children, sometimes because there was less refusal. Participants also reported variability in the groups of children and their eating behaviours, with some having ‘*bigger appetites*’ and being ‘*willing to try*’ which was perceived as easier; other groups were *‘pickier*’ and reported to be more challenging.

##### Parents’ expectations

Overall, participants felt comfortable approaching parents regarding their child’s feeding, working together and discussing feeding strategies with any concerns. It was typical for parents and educators to exchange information about the child’s food intake at home or during their time in the ELCC. Educators reported that some parents were more concerned than others about their child’s feeding, such as desiring that infants finish their bottles and requesting details in food diaries to monitor intake. This may have created an element of pressure on educators to engage in non-responsive behaviours as participants described sometimes rewarding the child for eating or avoiding feeding certain foods based on parental requests.

#### Physical opportunity

##### Mealtime characteristics

Responsive feeding seemed easier to support in some meals compared to others. For example, longer meals (e.g. lunch time compared to snack time) and using a family-style approach provided ECEs with more opportunities to sit with the children, engage in conversations and role modelling. During snack, an ‘open-snack’ approach allowed children to practice autonomy and decide when they were ready to come to the table, while still having some time parameters for when it was available. However, prior COVID-19 pandemic restrictions were described as preventing some mealtime characteristics and limited food-related play and exploration.

##### Centre location and availability of resources

Programme characteristics and available resources varied across ELCC and impacted responsive feeding. Connections with the community, proximity to local retailers (e.g. bakery and grocery store) and farms, or a space to grow vegetables in the centre (e.g. garden, raised beds and greenhouse) all provided opportunities for food exploration and facilitated food play outside of mealtime. Half of the directors spoke about challenges posed by the rising cost of food which limited some responsive feeding behaviours. For example, feeding the educators was perceived as difficult due to the cost of the food, which limited the opportunity to role model.

## Discussion

This study provides valuable insights into the influences on responsive feeding practices among ECEs and other staff in ELCC programmes across two provinces in Canada, through centring their social experiences within the COM-B model^(29)^. Our findings uncovered influences across all six COM-B domains providing a comprehensive understanding of responsive feeding behaviours and a potential for intervention across multiple COM-B domains which is essential for designing effective behaviour change strategies. Responsive feeding influences varied in their level of importance across the COM-B domains, meaning that some themes were more salient than others. For example, influences related to educators’ psychological capability, motivation and social opportunity were brought up more frequently compared to influences related to educators’ physical capability which included only a few mentions. This trend is consistent with similar research that excluded physical capability^(37)^ and might mean that this domain is less of a barrier in the ELCC context. Consideration of the influences across the COM-B domains within the context of the experience of ECEs will support intervention design to ensure the most relevant barriers are considered and addressed through implementation strategies.

### Capability

At the psychological-capability domain, we identified three influences that were consistently mentioned as affecting responsive feeding behaviours: participants’ lack of knowledge and skills for feeding children with challenging feeding patterns, difficulty distinguishing between pressure and encouragement, and managing the available food to ensure it is appropriately and fairly allocated according to children’s needs and desires. These challenges, predominantly practical in nature, underscore a noticeable gap in educator knowledge.

Refusal to eat, food neophobia and varying intake are common eating behaviours among young children and struggles of educators in ELCC programmes^(38–40)^. Similar to our findings, these types of behaviours can be triggering for educators, reduce their confidence to manage mealtimes and result in both responsive and non-responsive practices^(24)^. Additionally, encouraging children to try new foods is challenging, and ECEs often struggle to differentiate between encouragement and pressure. Ramsay et al.^(9)^ highlighted that ECEs exert pressure through verbal communication at mealtimes, not realising they may override children’s internal hunger and satiety cues^(9)^. Prompting the child to eat or engaging in negotiation were seen as positive strategies by educators when a child refuses to eat, rather than pressure^(26)^. Therefore, the current study reinforces the confusion that exists around what constitutes pressure and the need for further practical education and guidance in this area, especially given the knowledge that gets shared between educators and parents.

While nutrition education and training for educators can facilitate knowledge on responsive feeding behaviours^(28)^, existing interventions primarily emphasise nutrition literacy, along with the provision of healthy and unhealthy foods, rather than focusing on practical feeding strategies^(41–43)^. Lack of training on how to model healthful eating or support less adventurous eaters and clear guidelines on alternatives to pressure are typically missing from professional development^(22,23)^. All of our participating centres reported receiving minimal nutrition support (prior to participating in CELEBRATE Feeding) along with outdated nutrition guidelines in the respective provinces. Comprehensive training and technical assistance for educators could help them navigate challenging feeding situations, including managing the available food, working through food neophobia and encouraging (but not pressuring) food acceptance^(25,39,44)^. While nutrition education and training for educators can facilitate knowledge on responsive feeding behaviours^(28)^, existing interventions primarily emphasise nutrition literacy, along with the provision of healthy and unhealthy foods, rather than focusing on practical feeding strategies^(42,43)^. Lack of training on how to model healthful eating or support less adventurous eaters and clear guidelines on alternatives to pressure are typically missing from professional development^(22,23)^. All of our participating centres reported receiving minimal nutrition support (prior to participating in CELEBRATE Feeding) along with outdated nutrition guidelines in the respective provinces. Comprehensive training and technical assistance for educators could help them navigate challenging feeding situations, including managing the available food, working through food neophobia and encouraging (but not pressuring) food acceptance^(25,39,44)^.

### Motivation

Motivation is at the centre of the COM-B model and can directly influence the engagement of ECEs with responsive feeding behaviours^(29)^. In the current study, all ECEs had an overall goal to ensure that all children eat^(25)^; however, additional motivations and priorities at mealtimes varied, which reflect the diverse values that are shaped by cultural influence (e.g. table manners and utensil use), previous education and training, and their own experiences with food and feeding^(17,41,45)^. Some prioritised optimal nutrition, others focused on practical considerations and some aimed to create a social experience. When educators perceive themselves as role models to promote health and nutrition education, they employ various strategies at mealtimes to accomplish this^(26)^. Interestingly, both responsive and less responsive behaviours were reported concurrently, indicating that there is a spectrum of responsive feeding behaviours but also that motivation can change or also be influenced by capability and opportunity. For instance, an educator who prioritised mealtime as a social experience, which was often associated with responsive behaviours, may also resort to less-responsive communication under different circumstances, such as different children dynamics or rushed mealtime.

Furthermore, our study highlights the role of both reflective and automatic motivations in prompting educators to engage in responsive behaviours. Not only did educators want to see children enjoy themselves (*‘Mealtime “isn’t just about food”’*), but they also spoke of their own positive emotions and the joy they feel when they witness children’s happiness (*‘Enjoyment & fun’*), serving as automatic motivation. Educators can feel as the children’s proxy parents while at ELCC, genuinely caring about the child’s health and wellbeing^(39)^. Consequently, when children refuse to eat, they can feel discouraged in their role as a caregiver^(39)^. This underscores the complexity of understanding and maintaining the motivation of ECEs to engage in responsive feeding behaviours.

### Opportunity

Despite educators’ intentions to engage in responsive behaviours, the results of this study suggest they do not always have the social and physical opportunity. The leadership of ELCC directors has been previously reported as key to supporting their educators in changing nutrition practices in the centres^(16,44,46)^. Directors from the participating centres in this project had volunteered their programme for the CELEBRATE Feeding project, which demonstrates their commitment to supporting ECEs with implementing responsive feeding. This study further illustrated that although nutrition changes in ELCC can begin with the director’s vision, all roles (e.g. educators, cooks, inclusion support and the director) are needed to facilitate greater social opportunity and continuity for change^(16)^. Further, the results of our study pointed to the attention needed on enabling positive peer relationships during the process of change. Participants valued the relationships with their colleagues and expressed uncertainty in critiquing less responsive behaviour. This suggests the importance of focusing on enabling peer learning and collegiality in early childhood settings to create sustainable behaviour change^(47)^.

Additionally, many previous studies have indicated that educators often find their interactions with families challenging, particularly when it comes to discussing nutrition^(48–50)^. Educators can experience discomfort and lack self-efficacy to reach out to parents in fear of potentially criticising parents or delving into sensitive topics^(41,51)^. Participants in our study felt comfortable talking to parents and exchanging nutrition information; however, similar to other findings, they mentioned that parents are more likely to discuss nutrition just when they have concerns about the child’s eating^(48)^. Maintaining a positive relationship with parents is important for both ELCC staff and parents due to their reciprocal role in the development of children’s eating behaviours^(51)^. This dynamic can place demands on educators, eventually leading to pressure to please the parents^(25)^. Education, centre-level practices and policy empower educators, increasing their confidence in communicating with parents and legitimising their efforts to enforce nutrition changes^(41,48,50)^. Such measures, when incorporating responsive feeding, could extend educators’ communication with parents beyond food provision and dietary issues, facilitating the exchange of information about responsive feeding behaviours in a supportive, instructive and non-judgemental manner^(51)^.

In the realm of physical opportunity, centres varied in their characteristics and available resources. The type of meal (lunch or snack) and the setting (family-style or traditional serving) played a role in educators’ motivation to engage in responsive behaviours by providing or hindering opportunities. In this study, many participants mentioned that they would like to sit with the children and eat with them; however, they felt that other priorities and time constraints limited their opportunities. Some factors such as educator-child ratios, centre locations and COVID-19 restrictions were unlikely to be changed. However, other factors can be possibly addressed in future intervention through education and shifting participant priorities. First, allowing a flexible routine can minimise the need to rush mealtimes. If educators follow the children’s lead in transitioning from another activity to mealtime, children could come to the meal more prepared, enhancing their engagement with the food. Second, budgetary considerations can be reviewed to allocate more funds towards educator portions. Along with adjusting the menu to include their food preferences, this can encourage educators to enjoy meals with the children. Additionally, allowing children to serve themselves and take only what they want to eat can reduce food waste, potentially saving money. Adding physical resources, such as age-appropriate serving tools, will help to provide an enabling environment for children to serve themselves. Having an on-site garden and learning materials are also recognised as an effective strategy to involve children in nutrition education and encourage exploration of food^(20,26,44)^.

### Strengths and limitations

The use of reflexive thematic analysis was a strength of this research. While the initial coding process and theme development was conducted by one researcher, the final themes were discussed and finalised with the larger team. The research team all identify as women, some mothers, with expertise in paediatric nutrition and dietetics and/or early childhood. The reflexive approach applied by the team ensured a reflection on our assumptions informing data throughout the analysis process, attempting to keep meanings closer to participants’ ideas aligning with QD^(33)^ and the social constructivist paradigm^(34)^. Reflexive thematic analysis acknowledges the researcher’s role in knowledge production, and therefore, subjectivity is seen as an asset^(36)^. We acknowledge that this study described behavioural influences on responsive feeding, but the results may not be transferable to all ELCC contexts, and they were conducted at ELCC centres that had expressed an interest in responsive feeding. Although participants were encouraged to share their experiences to support intervention, their participation in the intervention was already secured; thus, there may have been a desirability bias by some participants. Additionally, not all ELCC staff participated in the interview process, which might also underrepresent some of the challenges to responsive feeding.

### Implications for practice

To date, this study is the first to employ the COM-B model to identify the factors influencing responsive feeding behaviours among ECEs in ELCC settings. Using this model provided a comprehensive understanding of the facilitators and obstacles to responsive feeding behaviours through all six COM-B domains recognising that behaviour is part of an interacting system of influences^(29)^. Further research could design an intervention by linking these influences with the BCW intervention functions^(29)^. For example, McIsaac et al. (2022) in their scoping review have identified education, training, environmental restructuring and enablement as the most influential intervention functions on implementation and sustainability of responsive feeding^(28)^. Based on the identified COM-B influences in this study, we suggest implications for practice to inform possible intervention strategies (Table 3) including critical supports to training and education to support capability and motivation alongside of other key functions to enable and sustain change. Future research from the CELEBRATE Feeding Study will describe implementation of behaviour change strategies to create sustainable change for responsive feeding in ELCC programmes.

**Table 2:** Summary of the identified influences on responsive feeding practices of ECEs within the six COM-B domains

COM-B domain	Definition (Michie et al., 2011)	Description	Influences (themes)	Illustrative quote
Capability (psychological)	Any mental process or skill that is required for the person to perform the behaviour (e.g. memory, attention and decision making abilities, knowledge)	Knowledge and skills to practice responsive feeding in challenging feeding situations	*Navigating feeding situations*	‘That’s when it gets a little tricky where they [children] really don’t want to have anything’. (Educator)
		Knowledge and capacity to encourage children to try new foods without pressure	*Balancing between pressure and encouragement*	‘I don’t know when to push, to say “you should try it” or if I should just let themselves figure out what they want. I do struggle with that because I want to do it right and I don’t know, because I do know it takes so many, like what is it, like 20 exposures over time or something before they’ll even try it’. (Educator)
		Knowledge around menu planning and resources management	*Managing available food*	‘I’m, of course, once again, most intrigued with the “support the children decide what and how much to eat.” I just find that piece fascinating. It’s just hard to wrap my head around it. They’re [children] all so different, and just trying to plan for that’. (Director)
Capability (physical)	Any set of physical actions that requires an ability or proficiency learned through practice	Ability to communicate with children that speak other language or have language difficulties	*Communication competence*	‘I find for my little group, language barriers are kind of difficult because I have a child in my group that I don’t understand what that child is trying to tell me…so that’s difficult to know what he wants when it comes to lunch time or snack time, that’s a challenge for sure’. (Educator)
		Ability to eat the same food as the children	*Dietary restrictions and educator preferences*	‘We do have some staff who have sort of issues - gluten free or, or dairy free or vegan, so they can bring their food to the table kind of thing’. (Director)
Motivation (reflective)	Plans, intentions, beliefs, identity	Educator approach to mealtime and/or to their role; Educator beliefs and attitudes towards food and feeding	*Mealtime ‘isn’t just about food’*	‘For me it’s just the conversation, the talking about the food, the sitting down talking about your day, what have you done, you know. It’s all about seeing the children and how they’re doing. If they’re not hungry, they’re not hungry’. (Director)
			*Healthy start/nourishment*	‘They [educators] encourage the children to try [the food], but it’s OK if you don’t, and we as a staff have just discussed that they don’t have to eat if they don’t want to, if they’re not hungry they’re not hungry, they have to listen to their—their own bodies’. (Director)
			*Ensuring intake*	‘Today we are having soup and grilled cheese sandwiches so I know some of them aren’t going to want the soup, but I’ll definitely offer them extras of the sandwiches’. (Educator)
Motivation (automatic)	Emotional, desires, impulses, habits	Educator’s emotions/desires that influence food and feeding, and automatic behaviours that result from feeding as a routine	*Enjoyment & fun*	‘One child in particular really liked the cherry tomatoes so we would come over, we’d pick them, she’d eat them, and it was just a nice little extra piece for the kids and to see it grow and then make something with it was really nice’. (Educator)
			*Managing mealtime and competing priorities*	‘As soon as we get the last one [child] served the first one wants more… so then we don’t get time to eat because we’re always serving the needs for the children—if it was in the middle [the food] they could just be like, “I want a little bit more of this and this” and they could serve it themselves’. (Educator)
Opportunity (social)	Opportunity afforded by interpersonal influences, social cues and cultural norms that influence the way we think about things	The relationships among educators, as well as between educators and children, influence feeding behaviours. This involves the extent to which educators feel supported and respected by their colleagues and the director in relation to food-feeding practices, along with the general environment and attitudes concerning food and feeding within the centre.	*Intra- and interprofessional dynamics*	‘There is a lot of respect around what you bring in but there is definite respect for—this is the plan, this is what’s best, this is best practice and that’s what we are going to do. So leave your belief about it at the door’. (Educator)
			*Child-Educator dynamics*	‘The tiny ones are not that good to tell, so sometimes I have to stop them when I see them out of food but my oldest one, he knows by himself when he is all done and so this is nice. He tells me, “all done” and so I make sure “so you don’t want anymore? You’re all done?” and he says “yes” and pushes his plate away’. (Educator)*** ‘I find that when I’m with the younger groups, I’m so relieved when I’m feeding them because I’m just like, “just eat it” and they will’. (Educator)
			*Parents expectations*	‘Parents are like, “oh well they eat it at home” and I’m like, “yeah” but I think there’s just sometimes there’s more distraction. That’s more like our younger kids right. So sometimes that can be a struggle for us like if they’re not gonna drink [the bottle] and I can’t force them obviously’. (Educator)
Opportunity (physical)	Opportunity afforded by the environment involving time, resources, locations, prompts/cues	Centre factors and meal characteristics	*Mealtime characteristics*	‘I would love to see going back to more family style. That’s when I found sitting down with them a lot more meaningful’. (Educator)
			*Centre location and availability of resources*	‘I also love being able to walk down to the experimental farm to the gardens there, we’ve done that before. Which is really nice to sort of bring the community aspect of like growing and food in’. (Educator)*** ‘We grow things we know they [children] can pick off the vine and eat raw. So they see it growing, they pick it, they wash—they see it being washed, and then cut up or just broken up and they eat it. Sometimes right out on the playground’. (Educator)

**Table 3:** Suggested intervention functions and strategies to build responsive feeding environment in ELCC centres

Training	Equip educators with practical strategies and train to react in responsive ways in challenging feeding situations. Educate on how to encourage food exploration and acceptance of food without pressure. Provide technical assistance with menu planning to ensure there is enough food and minimal waste
Education	Educate educators about different eating patterns and responsive language.
Enablement	Enable access to professional development opportunities such as workshops, seminars or online courses related to responsive feeding and nutrition practices.
Modelling	Provide educators with modelling opportunities where they can observe and learn from experienced colleagues in creating responsive feeding environments. Encourage mentorship programmes where experienced educators can guide newer staff in implementing these behaviours effectively.
Persuasion	Utilise a credible source (e.g. a dietitian) to persuade educators of the long-term benefits of responsive feeding, emphasising how it positively impacts children’s eating habits and overall wellbeing. Share success stories and case studies to illustrate the effectiveness of these behaviours. Provide reminders to educators of how well children are responding to changes and give examples of how outcomes may look if they continued with responsive feeding practices.
Incentivisation	Offer incentives in the form of funding for ELCC to invest in age-appropriate feeding tools, a mud kitchen or garden.
Restrictions	Draft centre-level policies that restrict using food as a reward or punishment; and encourage educators to sit with the children and eat the same food.
Environmental restructuring	Rearrange the physical environment to support responsive feeding, such as creating a calm and comfortable eating area. Ensure that there are age-appropriate and sufficient serving tools (e.g. bowls, tongs) to allow children to serve themselves.

### Conclusions

In conclusion, this study provides a comprehensive understanding of the influences on responsive feeding among ECEs within ELCC programmes across two Canadian provinces. The results from the interviews highlight that ECEs face numerous challenges in implementing responsive feeding practices, often balancing between promoting adequate intake and managing practical constraints, while also describing that many have the motivation needed to continue with responsive behaviours or change for the wellbeing of the children. All aspects of the COM-B were found to influence responsive feeding behaviours, where psychological capability, motivation and social opportunity were identified as key areas that require intervention. The findings provide evidence for the need of targeted support addressing these domains, such as enhanced training, education and resources in areas that focus on feeding strategies, managing food availability and distinguishing between pressure and gentle encouragement. Further, creating a supportive and collaborative environment among ECEs is important and fostering positive communication with parents is needed. Future research should focus on implementing and evaluating responsive feeding strategies and interventions to ensure their efficacy and practicality in unique ELCC settings. Investing in supporting ECEs to create a responsive feeding environment will help to support children develop a healthy relationship with food and contribute to their overall wellbeing.
